# A Cytotoxic and Anti-inflammatory Campesterol Derivative from Genetically Transformed Hairy Roots of *Lopezia racemosa* Cav. (Onagraceae)

**DOI:** 10.3390/molecules22010118

**Published:** 2017-01-12

**Authors:** Norma Elizabeth Moreno-Anzúrez, Silvia Marquina, Laura Alvarez, Alejandro Zamilpa, Patricia Castillo-España, Irene Perea-Arango, Pilar Nicasio Torres, Maribel Herrera-Ruiz, Edgar Rolando Díaz García, Jaime Tortoriello García, Jesús Arellano-García

**Affiliations:** 1 Centro Investigación en Biotecnología, Universidad Autónoma del Estado de Morelos, Av. Universidad 1001 Col, Chamilpa C.P. 62209, Cuernavaca, Morelos, Mexico; norma.moreno@uaem.mx (N.E.M.-A.); castillo@uaem.mx (P.C.-E.); iperea@uaem.mx (I.P.-A.); 2 Centro de Investigaciones Químicas-IICBA, Universidad Autónoma del Estado de Morelos, Av. Universidad 1001 Col, Chamilpa C.P. 62209, Cuernavaca, Morelos, Mexico; lalvarez@uaem.mx; 3 Centro de Investigación Biomédica del Sur (IMSS), Argentina No. 1, Xochitepec Centro C.P. 62790, Morelos, Mexico; azamilpa_2000@yahoo.com.mx (A.Z.); pisaliva@yahoo.com.mx (P.N.T.); edanae10@yahoo.com.mx (M.H.-R.); edgarrdg@hotmail.com (E.R.D.G.); jaime.tortoriello@imss.gob.mx (J.T.G.)

**Keywords:** genetic transformation, *Lopezia racemosa*, *Agrobacterium rhizogenes*, hairy roots, cytotoxic, anti-inflammatory

## Abstract

The genetically transformed hairy root line LRT 7.31 obtained by infecting leaf explants of *Lopezia racemosa* Cav with the *Agrobacterium rhizogenes* strain ATCC15834/pTDT, was evaluated to identify the anti-inflammatory and cytotoxic compounds reported previously for the wild plant. After several subcultures of the LRT 7.31 line, the bio-guided fractionation of the dichloromethane–methanol (1:1) extract obtained from dry biomass afforded a fraction that showed important in vivo anti-inflammatory, and in vitro cytotoxic activities. Chemical separation of the active fraction allowed us to identify the triterpenes ursolic (**1**) and oleanolic (**2**) acids, and (23*R*)-2α,3β,23,28-tetrahydroxy-14,15-dehydrocampesterol (**3**) as the anti-inflammatory principles of the active fraction. A new molecule **3** was characterized by spectroscopic analysis of its tetraacetate derivative **3a**. This compound was not described in previous reports of callus cultures, in vitro germinated seedlings and wild plant extracts of whole *L. racemosa* plants. The anti-inflammatory and cytotoxic activities displayed by the fraction are associated to the presence of compounds **1**–**3**. The present study reports the obtaining of the transformed hairy roots, the bioguided isolation of the new molecule **3**, and its structure characterization.

## 1. Introduction

Plants usually produce or accumulate very low quantities of metabolites of interest and large quantities of plant material are necessary to obtain an active substance, so in this sense hairy roots represent an alternative to overcome this problem and to produce higher amounts of secondary metabolites, or recently known specialized metabolites [1]. In recent years, several workers have reported the use of hairy root cultures for the production of secondary metabolites such as silymarin, a mixture of flavonolignans with hepatoprotective properties from hairy root cultures of *Sylibum marianum* [2], methyllycaconitine, a potential drug for the treatment of Alzheimer´s disease from mutagenesized hairy root cultures of *Solidago memoralis* [3], podophyllotoxin and 6-methoxy-podophyllotoxin from hairy roots of *Linum mucronatum* [4], and also the production of primary metabolites such as polyunsaturated fatty acids from hairy root cultures of *Echium acanthocarpum* [5]. Several additional strategies have also been implemented to enhance the metabolite production of hairy roots which include yield improvements as well as modification of the metabolism of hairy root cultures. Weathers et al. [6] reported that the addition of plant growth regulators could improve the growth and artemisinin production of hairy root cultures of *Artemisia annua*. Moreover, elicitors of secondary metabolism have long been used to increase the production of cell suspension and hairy root cultures [7].

**Figure 1:**
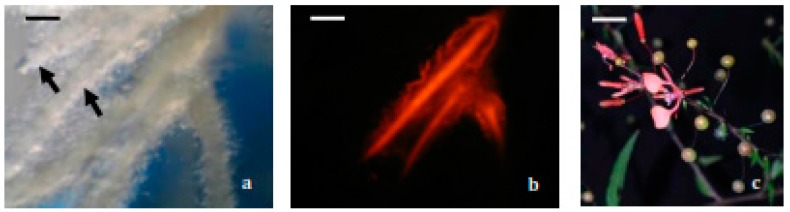
Photomicrographs (5×) of genetically transformed hairy roots of *Lopezia racemosa*. The same field observed under epifluorescence microscopy: (**a**) observed under white light; (**b**) observed under green light (550 nm). On the left upper part (arrows) non-fluorescent hairy roots derived from the same explant can be observed; (**c**) *Lopezia racemosa* wild plant. Scale bars: **a** and **b**: 1.0 mm; **c**: 1.0 cm.

*Lopezia racemosa* Cav. (Onagraceae) (Figure 1c), commonly known as punch herb or cancer herb, has long been used in traditional Mexican medicine to heal inflammatory diseases [8]. It was previously demonstrated that some fractioned extracts of this plant possess antimicrobial, antiparasitic, anti-inflammatory and cytotoxic activities [9]. Afterwards, bioassay-guided fractionation afforded the acylglucosylsterols 6-*O*-palmitoyl-3-*O*-β-d-glucopyranosylcampesterol (**I**) and 6-*O*-palmitoyl-3-*O*-β-d-glucopyranosyl-β-sitosterol (**II**), which were found to have anti-inflammatory and cytotoxic activities, respectively. Both were isolated and purified from wild plants, in vitro germinated seedlings, and callus cultures of *L. racemosa* [10]. Compound **II** was also isolated from *Ficus carica* [11]. Callus cultures produced lower or similar amounts of active metabolites compared to those found in the wild plant. In this study with the aim to increase the production of the anti-inflammatory and cytotoxic metabolites, transformed hairy roots lines from *L. racemosa* leaf explants were produced using *A. rhizogenes* strain ATCC 15834/pTDT. Selected hairy root line (LRT 7.31) showed in vivo anti-inflammatory activity and in vitro cytotoxic activity, but the phytochemical study outlined revealed that the active compounds **I** and **II**, previously described in the wild plant, in vitro germinated seedlings and callus cultures were not found, instead, a new sterol identified as (23*R*)-2α,3β,23,28-tetrahydroxy-14,15-dehydrocampesterol (**3**) was isolated and purified.

The present study describes the genetic transformation process and selection of hairy root lines, as well as the extraction, fractionation, and purification of the new sterol **3**, derived from the hairy root line LRT 7.31. Evaluation of the anti-inflammatory and cytotoxic activities is also discussed.

## 2. Results and Discussion

### 2.1. Hairy Roots Obtaining and Selection

From 113 infected leaf explants, only 43 generated hairy roots, giving a transformation frequency (TF) of 38%. The TF is usually reported as the percentage of explants that show a positive response relative to the total number of infected explants. The TF can vary widely with the plant species and *Agrobacterium* strain used, as well as with the conditions employed to carry out the infection and with the type of explants used, among others factors. In a recent report, Ashraf et al. [12] used the *A. rhizogenes* strain 15834 to infect leaf explants of *Persicaria minor* and obtained a TF of 8.8% using acetosyringone as an activator of *vir* genes. Wahyuni et al. [13], using the same *Agrobacterium* strain, obtained a TF of 70% in leaf explants of *Justicia gendarussa* without addition of *vir* gene inducers. Our results fall between the values mentioned above (38%).

After three weeks of bacteria elimination more than 300 individualized hairy roots derived from all the responsive explants were transferred to hairy root selection medium and 22 actively growing hairy roots were obtained, being LRT 7.31 one of the selected lines. The growth index (GI) of this line was 78.14 ± 3.14, resulting higher than other hairy root lines. It is important to note that this low number of transformed hairy roots could be explained by the fact that not all the roots emerging from an explant are transformed (Figure 1a,b).

**Figure 2:**
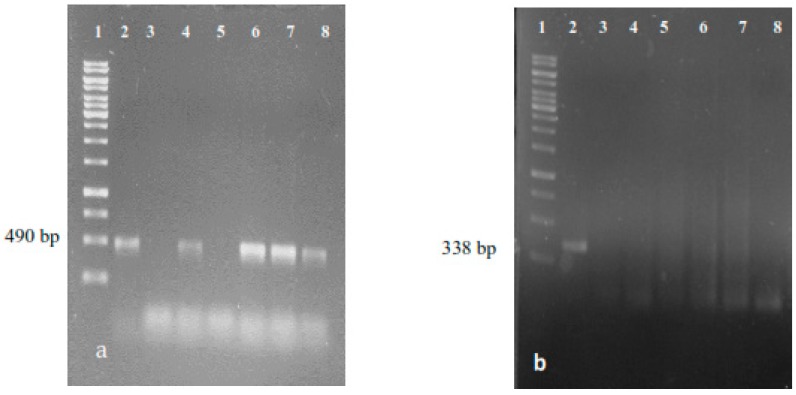
PCR products of transformed hairy roots derived from *L*. *racemosa*. (**a**) Amplifying a 490 bp fragment of the *rolC* gen of *A. rhizogenes* ATCC 15834. Lane 1: 1 Kb DNA marker; lane 2: Positive control, PCR product from total DNA of *A. rhizogenes* ATCC 15834; Lane 3: Negative control, PCR product from total DNA of non-transformed roots of *L. racemosa*; lanes 4–8: PCR products from total DNA of 5 hairy root lines derived from *L. racemosa*, (lines 7.31, 6.14, 6.4, 3.1 and 17.6, respectively); (**b**) Amplifying a 338 bp fragment of the *virD* gen of *A. rhizogenes* ATCC 15834. Lane 1: 1 Kb DNA marker; lane 2: Positive control, PCR product from total DNA of *A. rhizogenes* ATCC 15834; lane 3: Negative control, PCR product from total DNA of non-transformed roots of *L. racemosa*; lanes 4–8: PCR products from total DNA of 5 hairy root lines derived from *L. racemosa*.

The genetic transformation of selected hairy root lines such as LRT 7.31, LRT 6.14, LRT 6.4, LRT 3.1 and LRT 17.6 was confirmed by PCR analysis (Figure 2). We observed that among the tested root lines, only line LRT 6.14 did not amplify the expected fragment of 490 bp, although it is actively growing. It is necessary to test amplification for other *rol* genes, such as *rolB* or *rolA*, in order to explain the active growth of this hairy root line.

It had been proposed that crown galls induced by *A. tumefaciens* are chimerical tissues, since the auxin and cytokinin produced by transformed plant cells containing and expressing the genes transferred through the T-DNA modify the growth pattern of untransformed cells beside them, while roots induced by *A*. *rhizogenes* appear to be composed only of transformed cells [14]. However, we found that in our case most of the roots emerging from the responsive explants did not continue growing when they were cut off from the original explant and individually transferred to a new culture medium without plant growth regulators. We therefore considered each individual hairy root as a product of different transformation events, since we observed at least three different root phenotypes: red fluorescent non-actively growing roots, non-fluorescent actively growing roots and red fluorescent actively growing roots. This could be explained by the fact that the *A. rhizogenes* strain used in this study carries two plasmids, in which the wild type Ri plasmid contains in its TL-DNA the *rol* genes responsible to induce the phenotype “hairy root”, while the binary vector that possesses on its T-DNA the gene encoding for the red fluorescent protein, responsible for the phenotype “red fluorescent”. In this sense and in order to have the last phenotype of hairy roots mentioned above, a simultaneously occurring double T-DNA transference event was necessary. The other phenotypes are explained by single transference events. On the other hand, line LRT 6.14, which did not amplify the expected fragment corresponding to the *rolC* gene, can be explained as an incomplete transference event or by rearrangements and deletions that for long time have been known to occur with some frequency during the process of T-DNA transference and integration [15,16,17,18].

### 2.2. Anti-Inflammatory and Cytotoxic Activity of the Transformed Line LRT 7.31

The CH_2_Cl_2_:CH_3_OH (50:50 *v*/*v*) crude extract obtained from the LRT 7.31 line was tested in the mouse ear edema induced with 12-*O*-tetradecanoylphorbol-13-acetate (TPA) technique and compared with previous reports as well as with fractioned extracts. Our results showed that the crude extract inhibited by 50.5% ± 6.06% (mean ± standard deviation: SD) the inflammation at a dose of 1 mg/ear, with median Inhibitory Concentration (IC_50_) of 1.2 mg/ear. This value was similar with that previously obtained by Salinas et al. from crude extracts of wild plants, in vitro seedlings and callus cultures of this species (58.3% ± 1.73% of inhibition) [9]. Chromatographic purification of the crude extract afforded four main fractions, of which C1F3 inhibited the TPA-induced inflammation of the mouse ear by 85.6% ± 9.08% (Mean ± SD) at a dose of 1 mg/ear (IC_50_ 0.28 mg/ear), higher than the crude extract, and similar than that displayed by the positive control Indomethacin (85.7% ± 6.47%: Mean ± SD) at the same dose.

Regarding to cytotoxicity, the crude extract also showed cytotoxic activity against HCT-15 (colon adenocarcinoma) and OVCAR (ovary carcinoma) with IC_50_ values of 3.14 μg /mL and 0.57 μg/mL respectively, but non active against HeLa (cervical carcinoma) and KB (laryngeal carcinoma) cancer cell lines (Table 1), whereas the C1F3 fraction showed to be highly active against HeLa and KB cancer cell lines as compared with the crude extract, and still active against HCT-15 and OVCAR. The IC_50_ values were 0.00089 μg/mL, 5.39 μg/mL, 3.32 μg/mL and 3.069 μg/mL, respectively (Table 1).

**Figure 3:**
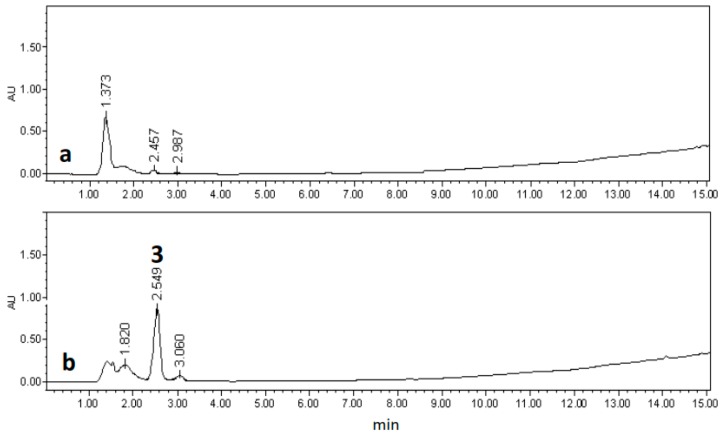
HPLC profiles of (**a**) CH_2_Cl_2_:CH_3_OH extract and (**b**) Fraction C1F3 obtained from hairy root line LRT 7.31.

Fraction C1F2 did not induce any anti-inflammatory effect and its chemical analysis indicated the presence of the mixture of the triterpenes ursolic (**1**) and oleanolic (**2**) acids which were identified by ^1^H- and ^13^C-NMR spectral data [19] as well as by direct comparison with authentic samples available in our laboratory [20]. Although compounds **1** and **2** have been already described to possess weak anti-inflammatory activity, it is likely that their poor solubility inhibited their detection in our model. Fraction C1F3 was subjected to successive chromatographic purification, obtaining an amorphous solid which probed to be a mixture by NMR and HPLC analyses. The compound **3** present in the crude extract was quantified, being 28.9 μg/mg, while in the fraction C1F3 it was 850.0 μg/mg. The HPLC profile of fraction C1F3 (Figure 3) indicated that compound **3** is the principal component in the fraction (85%) and probably the responsible of the anti-inflammatory and cytotoxic activities displayed by this fraction.

**Figure 4:**
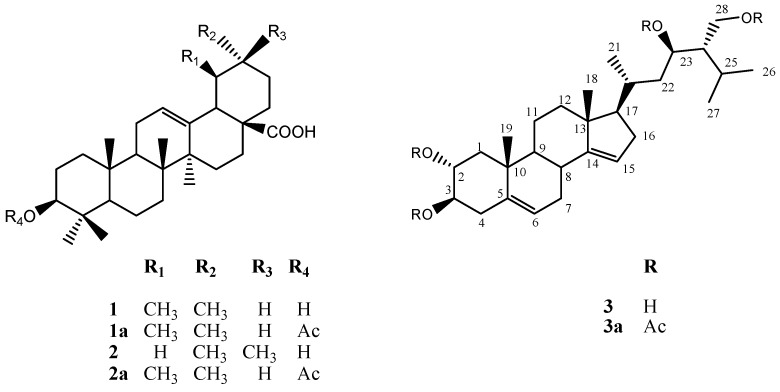
Chemical structure of compounds **1**, **1a**, **2**, **2a**, **3** and **3a** isolated from line LRT 7.31 hairy roots of *L. racemosa*.

With the aim of identifying this compound, fraction C1F3 was acetylated and further chromatographic purification allowed the isolation of acetylursolic acid (**1a**) and acetyloleanolic acid (**2a**) as the minor constituents of the fraction, while the new steroid identified as (23*R*)-2α,3β,23,28-tetraacetyl-14,15-dehydrocampesterol (**3a**) was the major constituent of this fraction (Figure 4).

### 2.3. Chemical Characterization

Compound **3a** showed a pseudomolecular ion [M − H]^−^ peak at *m*/*z* 613.3819 by HRFABMS in the negative mode, corresponding to a molecular formula C_36_H_54_O_8_ which was also deduced on the basis of the ^13^C-NMR spectrum combined with DEPT data. Assignment of all the ^13^C- and ^1^H-NMR signals for each spin systems is shown in Table 2. The ^1^H- and ^13^C-NMR data indicate that derivative **3a** contains the C-28 tetracyclic steroidal system ring of campesterol [δ 1.16 (3H, s, H-18), 1.21 (3H, s, H-19), 0.81 (3H, d, *J* = 6.4 Hz, H-21), 0.87 (6H, d, *J* = 7.6 Hz, H-26, H-27)].

**Figure 5:**
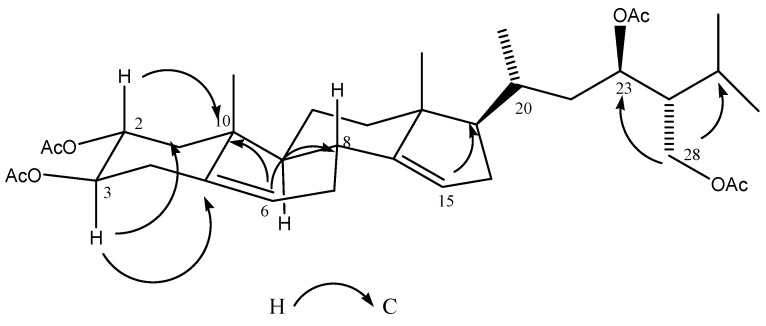
Key HMBC correlations of derivative **3a**.

The ^1^H-NMR spectrum of compound **3a** displayed signals from the protons of two tetrasubstituted double bonds at δ 5.24 (1H, t, *J* = 6.8 Hz) and δ 5.22 (1H, t, *J* = 7.2 Hz), three acetoxy methine groups at δ 5.05 (2H, ddd, *J* = 3.2, 10.4 Hz), and 5.14 (1H, ddd, *J* = 4.4, 10.4, 11.6 Hz), and the AB system of an acetoxy methylene group at δ 3.80 (1H, dd, *J* = 3.6, 11.6) and δ 3.55 (1H dd, *J* = 1.2, 12.0 Hz). The ^13^C-NMR spectrum of **3a** showed 28 signals (5 CH_3_, 8 CH_2_, 11 CH, and 4 C) corresponding to the steroid skeleton, as well as the signals for the four acetates present in the molecule (δ 170.72/21.53, 170.54/21.02, 170.54/20.80 and 170.42/20.77). A detailed analysis of NMR data (COSY, HSQC, and HMBC) revealed the absence of the methyl group at C-24 having instead an acetoxy methylene evidenced by the AB system at δ 3.80 and 3.55, and the observed correlations between the methylene protons at C-28 with C-23 (δ 75.55) and C-25 (δ 23.79) in the HMBC spectrum (Figure 5), indicating also the presence at C-23 of one of the three acetoxy methines deduced early. Comparison of the chemical shift and *J* values of H-23 (δ 5.5, dd; *J* = 3.2, 10.4 Hz) with those described for related C-23 oxygenated sterols [21] allowed us to establish the C-23*R* configuration of **3a**.

The remaining acetoxymethines at δ 5.05 and 5.14 were located at C-3 and C-2, respectively, by the HMBC correlations between H-2 (δ 5.14) with C-1 (δ 39.92) and C-10 (δ 31.38), and between H-3 (δ 5.05) with C-4 (δ 31.43) and C-5 (δ 139.52). Likewise, HMBC correlations of H-2, H-3, H-23 and H-28 with their corresponding carbonyl esters were observed (Table 2). The α-configuration of the C-2 acetoxyl group was evident from the chemical shift and the *J* values of this proton (δ 5.14, ddd, *J* = 4.4, 10.4, 11.6 Hz) compared with related compounds [22,23]. The ^1^H-^13^C ^3^J correlations between the vinylic protons at δ 5.24 with C-8 (δ 38.63) and C-10 (δ 31.38), and between δ 5.22 with C-13 (δ 46.84) and C-17 (δ 53.96) allowed us to locate the double bonds at C-6 and C-15 respectively (See Supplementary Data). On the basis of all these evidences, the natural product was identified as (23*R*)-2α,3β,23,28-tetrahydroxy-14,15-dehydrocampesterol (**3**), a new natural product.

## 3. Experimental Section

### 3.1. General Procedures

Compounds were isolated by means of open column chromatography (CC). Analytical TLC was carried out on precoated silica gel 60F254 plates (Merck, Darmstadt, Germany). All NMR spectra and two-dimensional spectroscopy experiments COSY, HSQC, HMBC were recorded on an INOVA-400 instrument (Varian, Palo Alto, CA, USA) at 400 MHz for ^1^H-NMR spectra in CDCl_3_ with tetramethylsilane (TMS) as internal standard and at 100 MHz for ^13^C-NMR. Chemical shifts are reported in δ values. High resolution mass spectrometry in negative ion mode (HRFABMS) was performed using an AX 505 HA (JEOL, Tokyo, Japan) mass spectrometer. The IR spectrum was recorded on a Tensor 27 FTIR (Bruker, Fremont, CA, USA). Melting points were determined on a Fisher-Johns Melting Point apparatus. Optical rotations were measured on a 241 digital polarimeter (PerkinElmer, Waltham, MA, USA) equipped with a sodium lamp (589 nm) and a microcell. High pressure liquid chromatography was performed using a Waters Delta prep 4000 chromatograph equipped with a Waters 717 plus Autosampler and 996 photodiode array detector (Waters Co., Milford, MA, USA), and a Chromolith Performance C18 column (5 μm, 7.8 mm × 100 mm). The analysis was run with a gradient system of solvent A (H_2_O:CH_3_CN) on solvent B (CH_3_CN), UV detection at 205 nm at a flow rate of 1.0 mL/min, and using 20 μL sample injection.

### 3.2. Bacterial Strain

The strain of *A*. *rhizogenes* ATCC15834/pTDT [24] was grown over 48 h at 28 ± 1 °C in liquid yeast-mannitol (YM) broth containing spectinomycin 100 mg/L, reaching an optical density (OD) of 0.4–0.6 at 600 nm. 

### 3.3. Plant Material

The seeds of *L*. *racemosa* used in the present study were obtained from the stock reported by Salinas et al. [9].

### 3.4. Seed Germination and Axenic Seedling Obtaining

Seeds were surface sterilized in small filter paper bags dipped in a solution of domestic liquid detergent and sterile distilled water 1% (*v*/*v*) for 10 min. followed by 1 min. in 70% (*v*/*v*) ethanol and 15 min in 15% commercial bleach (6% active chlorine). Finally, seed bags were rinsed five times in abundant sterile distilled water. Once sterilized, the seeds were transferred to small glass jars containing half strength salts and organic components of Murashige and Skoog [25] (MS) medium, with no plant growth regulators, 3% sucrose and 0.3% Gelzan™ CM^®^ (Sigma-Aldrich, Co., St. Louis, MO, USA); pH adjusted to 5.6 ± 0.1 and autoclaved at 108 kPa and 121 °C for 20 min. The glass jars were then transferred to a growth chamber and incubated at 25 ± 1 °C with a photoperiod of 16/8 h light/darkness and 27 μmol·m^2^·s^−1^ white photon flux density.

### 3.5. Genetic Transformation

#### 3.5.1. Hairy Root Induction

Leaf explants prepared from 30-day-old aseptic seedlings germinated in vitro were used for infection with the *A*. *rhizogenes* strain described above. One milliliter of bacterial liquid culture was mixed with 10 mL of sterile distilled water in a Petri dish. The previously prepared leaf explants were then dipped in this diluted bacterial suspension and incubated for 15 min. The explants were transferred to Petri dishes containing MS/B5 co-culture medium with macro and micro nutrients of the MS medium and vitamins of the Gamborg et al. [26] B5 medium, 3% sucrose, 0.3% Gelzan™ CM^®^, with no plant growth regulators, pH adjusted to 5.6 ± 0.1 and autoclaved at 108 kPa and 121 °C for 20 min. A control was established containing non-infected explants. Ten explants per Petri dish containing the co-culture medium were transferred. Co-culture was carried out for 48 h under the same environmental conditions used during seed germination. The explants were then rinsed three times in sterile distilled water containing 500 mg/L ticarcillin and 200 mg/L cefotaxime antibiotics in order to begin the *Agrobacterium* elimination. The rinsed explants were then placed once again on sterile filter paper and transferred to a fresh co-culture medium containing the same antibiotics (200 mg/L of each) and incubated under the same environmental conditions described above.

#### 3.5.2. Selection and Establishment of Hairy Root Lines

Emerged hairy roots from explants infected with *Agrobacterium* were observed under an epifluorescence microscope (Carl Zeiss V8) and microphotographs were taken. Each hairy root emerged from each explant was removed and individually cultivated in a new Petri dish containing full-strength co-culture MS/B5 medium, with no antibiotics and under the same environmental conditions described above. For selection purposes, each hairy root was classified and identified with the number assigned to its explant and another consecutive number, such that each root could be clearly identified and putatively considered as a root line. Two criteria were used for selection: growth rate and fluorescence. Growth rate was determined for line: LTR-7.31. After six months in culture, these lines produced enough biomass to enable estimation of their GI, and 3 replicates of fresh weight 0.5 g and a control for initial dry weight were inoculated and cultivated for 30 days in glass jars containing the same culture medium. They were then weighed to determine their fresh weight and dried at room temperature in order to record dry weight. Growth rate was determined as reported by Ashraf et al. [12], calculating the growth index using the following formula: GI = rwf − rwi/rwi; where rwf is final root weight and rwi is initial root weight.

### 3.6. DNA Isolation and PCR Analysis

#### 3.6.1. DNA Isolation

Genomic DNA was isolated from fresh plant material of diverse selected hairy root lines and also from non-transformed roots of *L*. *racemosa*, using the kit ZR Plant/Seed DNA MicroPrep™ (Zymo Research, Irvine, CA, USA). A total of 150 mg of each sample were finely cut and placed in a lysis tube, adding 750 μL of lysis solution. Each sample was then agitated for 10 min in a vortex at maximum speed in order to disrupt the tissue. The lysis tube was then centrifuged at 10,000 × *g* for 1 min. Following centrifugation, 400 μL of supernatant was transferred to a filter tube connected to a collector tube and centrifuged once again at 7000 × *g* for 1 min. All of the following steps were carried out according to the protocol of the kit manufacturer.

#### 3.6.2. PCR Analysis

DNA samples of each hairy root were used as template for PCR analysis to determine the presence of the *rolC* gene in transformed hairy roots and the absence of *virD2* in the same roots using specific primers reported by Bonhomme et al. [27]: 5′TGTGACAAGCAGCGATGAGC3′ and 3′GATTGCAAACTTGCACTCGC5′ as well as 5′ATGCCGATCGAGCTCAAGT3′ and 3′CCTGACC CAAACATCTCGGCTGCCA5′. The first pair was designed to amplify a 490 bp fragment of the *rolC* gene, and the second to amplify a 338 bp fragment of the *virD2* gene, which is used as a control since it is not transferred to the plant cell during the transformation process. A DNA amplification kit from Vivantis was used. Each sample was prepared in 500 μL PCR tubes on ice in order to obtain a total reaction volume of 50 μL, comprising 1 μL DNA template, 5 μL 10X Taq DNA polymerase reaction buffer, 2 μL 50 mM MgCl2, 1 μL of each primer (10 μM), 2 μL dNTPs mixture (2 mM), 0.5 μL of recombinant Taq DNA polymerase (5 μ/μL) and 37.5 μL of nuclease free water. PCR amplification for both genes was carried out in an Mastercycler Gradient (Eppendorf, Hamburg, Germany) device under the following conditions: 1 cycle of 5 min at 95 °C, 35 cycles of 1 min denaturing at 95 °C, 1 min annealing at 50 °C, and 1 min extension at 72 °C; finally, 1 cycle of 5 min final extension at 72 °C. The PCR products were subjected to electrophoresis in 1% agarose gel at 100 volts for 60 min and visualized on UV transilluminator (BioDoc-It™ Imaging System, Upland, CA, USA) using ethidium bromide staining and photographed.

### 3.7. Extraction and Isolation of Chemicals Compounds from of Selected Hairy Root Line LTR-7.31

The plant material (84 g) from the in vitro culture (LTR-7.31) was dried, pulverized and extracted with CH_2_Cl_2_:CH_3_OH 50:50 (*v*/*v*) via maceration at room temperature for 72 h (840 mL × 3). The liquid extract was filtered using No. 1 Whatman filter paper and concentrated to dry in a Büchi-490 rotary evaporator (Büchi, Flawil, Switzerland) at 40 °C under low pressure. Final extract (5.7 g) was stored at 4 °C for later chromatographic and pharmacologic analysis. The extract was fractionated by column chromatography (silica gel 60, Merck) eluting with a gradient system of *n*-hexane:ethyl acetate (100:00 → 00:100) to afford four fractions: C1F1, 540 mg (90:10), C1F2, 382 mg (80:20), C1F3, 113 mg (70:30), and C1F4, 422 mg (50:50). Further chromatography of fraction C1F2 over silica gel eluted with *n*-hexane:ethyl acetate (9:1), gave the natural mixture of the triterpenic acids ursolic (**1**) and oleanolic (**2**). C1F3 (60 mg) was acetylated with acetic anhydride (2 mL) and pyridine (1 mL) for two hours, the product dissolved in dichloromethane was purified by column chromatography with silica gel 60 using a gradient of *n*-hexane:EtOAc (100:00 → 70:30) to give 8 mg of the mixture of 3-acetyl ursolic acid (**1a**) and 3-acetyl oleanolic acid (**2a**) and 24 mg of the new compound (23*R*)-2α,3β,23,28-tetraacetyl-14,15-dehydrocampesterol (**3a**). Amorphous solid; [α]${}_{D}^{20}$ + 15.4 (*c* 0.8 CHCl_3_); IR (KBr)_νmax_ 2962, 1740, 1468, 832 cm^−1^; ^1^H (CDCl_3_, 400 MHz) and ^13^C (CDCl_3_, 100 MHz) NMR data see Table 2; FABMS *m*/*z* 613 [M − H]^+^; HRFABMS (negative) *m*/*z* [613.3819 [M − H]^−^ (calcd for C_36_H_54_O_8_)].

### 3.8. Antiinflammatory and Cytotoxic Activities

#### 3.8.1. TPA Induced Mice Ear Inflammation Model

This assay was used to evaluate the anti-inflammatory activity of the plant extracts. Male ICR mice between 25 and 30 g in weight were maintained under standard laboratory conditions (12/12 h light/darkness, 25 °C ± 3 °C temperature, 70% ± 5% relative humidity, with food and water *ad libitum*). All procedures were conducted according to the Official Mexican Rule NOM-062-ZOO-1999 (technical specifications for the production, care, and use of laboratory animals) and international ethical guidelines for the care and use of experimental animals. The extracts were dissolved in methanol:acetone 50:50 *v*/*v* (vehicle) to a final concentration of 50 mg/mL. Mice were divided into 4 groups of 6 mice each; one group was used as negative control (vehicle), another was used to compare the anti-inflammatory drug indomethacin (positive control) with the other groups of tested hairy root extracts. To evaluate testing groups, 10 μL of each extract was applied to the inner and 10 μL to the outer surface of the right ear (1 mg/ear) of each mouse; the left ear was treated in the same form, but the inner and outer surface were treated with 10 μL of vehicle. For the indomethacin (Sigma-Aldrich, Co.) group, 20 μL of vehicle was used to apply the compound on the right ear (1 mg/ear) of each mouse and for the 12-*O*-tetradecanoylphorfol-13-acetate (TPA, Sigma-Aldrich, Co.) control group, only vehicle was used in both ears. After 10 min, 2.5 μg of TPA was applied to the right ear of all groups in order to induce ear inflammation. Four hours after the application of TPA, the mice were sacrificed in a chloroform chamber followed by cervical dislocation. Finally, circular sections of 6 mm in diameter were taken from the central part of both the treated (tr) and non-treated (nt) ears of each mouse. Each section was weighed to determine the inflammation percentage by weight difference between the treated and non-treated ear. Percentage of inflammation inhibition was calculated using the following formula: Inhibition % = [∆w control − ∆w extract treated/∆w control] [100] where ∆w = wtr − wnt; with wtr representing the weight of the section of the treated right ear and wnt the weight of the section of non-treated left ear. A curve of effect-concentration with five different amounts of extract (0.25, 0.5, 0.75, 1.0 and 1.5 mg/ear) was generated for the most active extract.

#### 3.8.2. Cytotoxic Activity

In order to evaluate the cytotoxic activity of the extracts derived from the hairy root cultures, cell lines of different human cancer types such as: HeLa (Cervical carcinoma), HCT-15 (Colon adenocarcinoma), OVCAR (Ovary carcinoma) and KB (Laryngeal carcinoma) were used. Cell lines were cultivated in Eagle´s Minimum Essential Medium (MEM) containing 10% Fetal Bovine Serum (FBS), under the following conditions: 37 °C temperature, 5% atmospheric CO_2_ and 100% relative humidity. Cell lines in log phase were treated with 3 different concentrations of each extract: 1, 10, and 100 μg/mL, and incubated for 72 h under the same environmental conditions. Cell concentration was calculated by protein analysis. Results are expressed as the concentration that inhibits growth by 50% over the incubation period (IC_50_). Values are estimated from semi-log concentration (μg/mL), versus percentage of viable cells.

## 4. Conclusions

Through a process of genetic transformation and careful selection of hairy roots, we established 22 different lines of actively growing hairy root cultures. From the results of the anti-inflammatory and cytotoxic activities of CH_2_Cl_2_:CH_3_OH extracts derived from the selected line, we conclude that line 7.31 possesses high anti-inflammatory and cytotoxic activities. In the extract we did not find the compounds which were reported previously by Salinas et al. [11], however, we isolate and characterized a novel compound (23*R*)-2α,3β,23,28-tetrahydroxy-14,15-dehydrocampesterol as the major component in the fraction C1F3. It will be necessary to continue with the pharmacological characterization of this major component of the fraction C1F3 in the future. The pharmacological characterization can be done by (a) its further purification, (b) testing its anti-cytotoxic activity in normal human fibroblasts and (c) studying its action mechanism by flux cytometry. Regarding the hairy root line LRT 7.31, it will be necessary to study its growth kinetics and its metabolite production, which can be done by batch culture in liquid medium. Finally, it is important to mention that the present study is the first report about genetic transformation of this plant species.

## Figures and Tables

**Table 1:** Cytotoxicity (IC_50_) of CH_2_Cl_2_:CH_3_OH crude extract and fraction C1F3 from hairy root line LRT 7.31 and wild plant extract of *L. racemosa*.

Sample Origin	Cancer Cell Lines			
	HeLa (μg/mL)	HCT-15 (μg/mL)	OVCAR (μg/mL)	KB (μg/mL)
Crude extract	63.97 ± 0.09	3.14 ± 0.03	0.57 ± 0.01	>100
C1F3	0.00089 ± 0.000098	3.32 ± 0.03	3.069 ± 0.01	5.39 ± 0.12
Wild plants	>100	5.6 ± 0.11	0.08 ± 0.03	>100

HeLa: Cervical carcinoma; HCT-15: Colon adenocarcinoma; OVCAR: Ovary carcinoma; KB: Laryngeal carcinoma.

**Table 2:** ^1^H (400 MHz) and ^13^C (100 MHz) NMR data for compound 3a (acetone-*d_6_*).

Position	δ_H_	δ_C_	Type
1	1.31, 2H, m	39.92	CH_2_
2	5.14, 1H, ddd (4.4, 10.4, 11.6)	70.29	CH
3	5.05, 1H, ddd (3.2, 10.4)	75.55	CH
4	1.76, 2H, m	31.43	CH_2_
5	------------	139.52	C
6	5.24, 1H, t (6.8)	122.72	CH
7	1.74, 2H, m	28.83	CH_2_
8	1.82, 1H, m	38.63	CH
9	1.68, 1H, m	48.45	CH
10	------------	31.38	C
11	1.38, 2H, m	23.86	CH_2_
12	1.32, 2H,m	34.53	CH_2_
13	----------	46.84	C
14	----------	145.19	C
15	5.22, 1H,t (7.2)	125.89	CH
16	1.72, 2H, m	33.46	CH_2_
17	1.72, 1H, m	53.96	CH
18	1.16, 3H, s	24.14	CH_3_
19	1.21, 3H, s	24.24	CH_3_
20	1.44, 1H, c	33.14	CH
21	0.81, 3H, d (6.4)	17.72	CH_3_
22	1.38, 2H, m	37.65	CH_2_
23	5.05, 1H, ddd (3.2, 10.4)	75.55	CH
24	1.24, 1H, m	44.41	CH
25	1.76, 1H, m	23.79	CH
26	0.87, 3H, d, (7.6)	17.43	CH_3_
27	0.87, 3H, d (7.6)	17.58	CH_3_
28	3.80, 1H, dd, (3.6, 11.6) 3.55, 1H, dd, (1.2, 12)	65.93	CH_2_
CH_3_-CO-	1.93, 3H, s	20.77	CH_3_
	1.93, 3H, s	20.80	CH_3_
	1.99, 3H, s	21.02	CH_3_
	2.02, 3H, s	21.53	CH_3_
CH_3_-CO-		170.42	C
		170.54	C
		170.54	C
		170.72	C

## Supplementary Materials

Supplementary materials can be accessed at: http://www.mdpi.com/1420-3049/22/1/118/s1.
