# Supplementary material for: A Cytotoxic and Anti-inflammatory Campesterol Derivative from Genetically Transformed Hairy Roots of Lopezia racemosa Cav. (Onagraceae)
Source: Molecules. 2017 Jan 12;22(1):118. doi: 10.3390/molecules22010118 (PMC6155711; doi:10.3390/molecules22010118)
Supplement: Supplementary file 1 [file molecules-22-00118-s001.pdf]

# Supplementary Materials: Cytotoxic and Anti-Inflammatory Campesterol Derivative from Genetically Transformed Hairy Roots of *Lopezia racemosa* Cav. (Onagraceae)

**Norma Elizabeth Moreno-Anzúrez, Silvia Marquina, Laura Alvarez, Alejandro Zamilpa, Patricia Castillo-España, Irene Perea-Arango, Pilar Nicasio Torres, Maribel Herrera-Ruiz, Edgar Rolando Díaz García, Jaime Tortoriello García and Jesús Arellano-García**

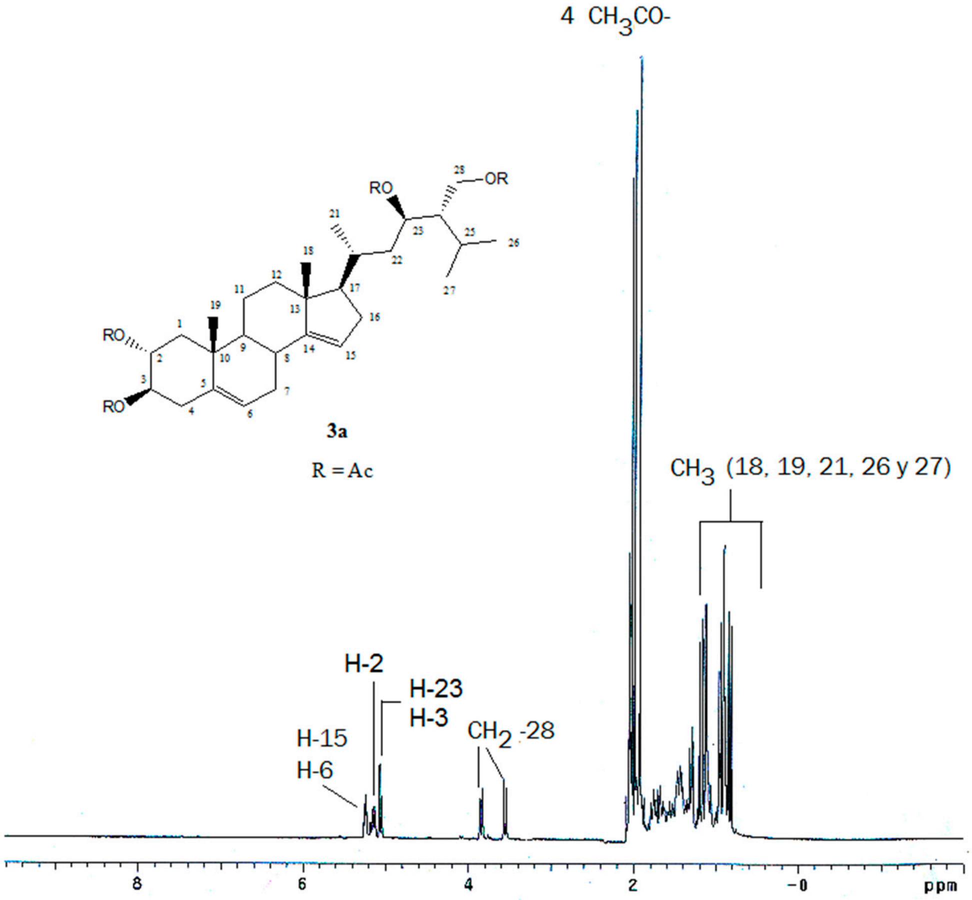


**Figure S1.** $^{1}$H-NMR (400 MHz; acetone-$d_6$) of compound (23*R*)-2α,3β,23,28-tetraacetyl-14,15-dehydrocampesterol (**3a**).

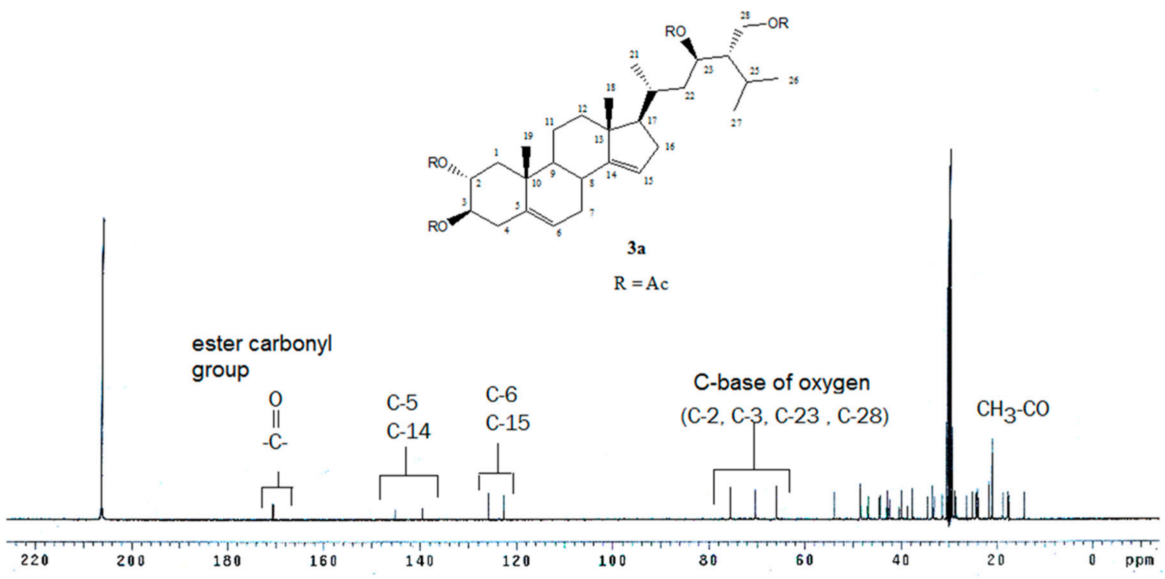


**Figure S2.** $^{13}$C-NMR (100 MHz; acetone-$d_6$) of compound (23*R*)-2α,3β,23,28-tetraacetyl-14,15-dehydrocampesterol (**3a**).

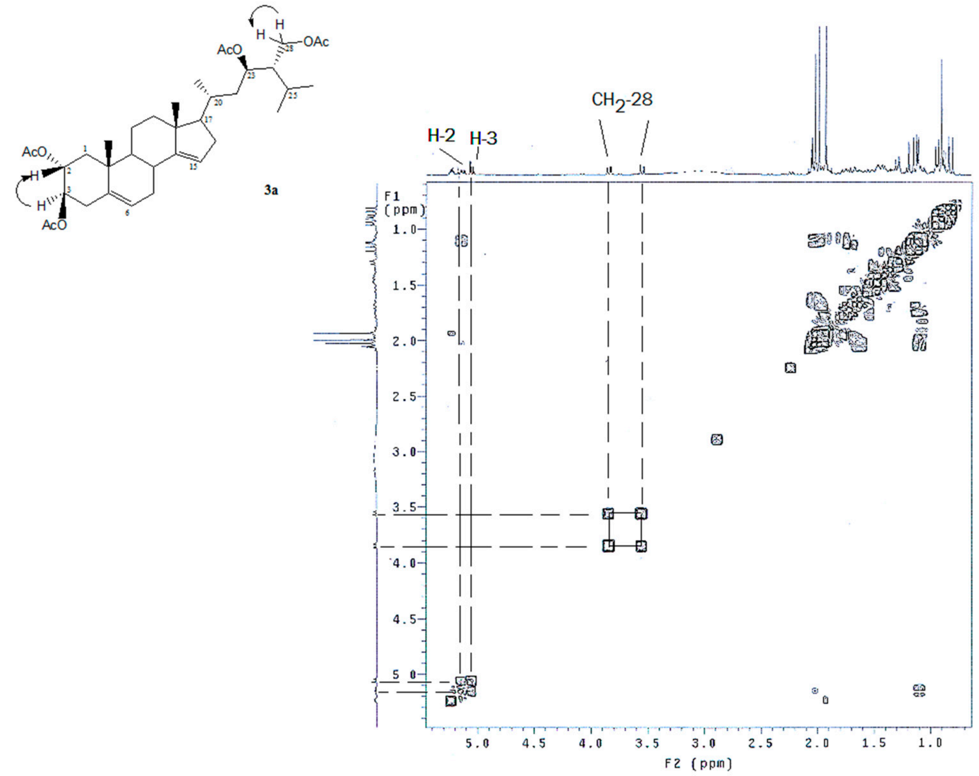


**Figure S3.** COSY (400 MHz; acetone-$d_6$) of compound (23*R*)-2α,3β,23,28-tetraacetyl-14,15-dehydrocampesterol (**3a**).

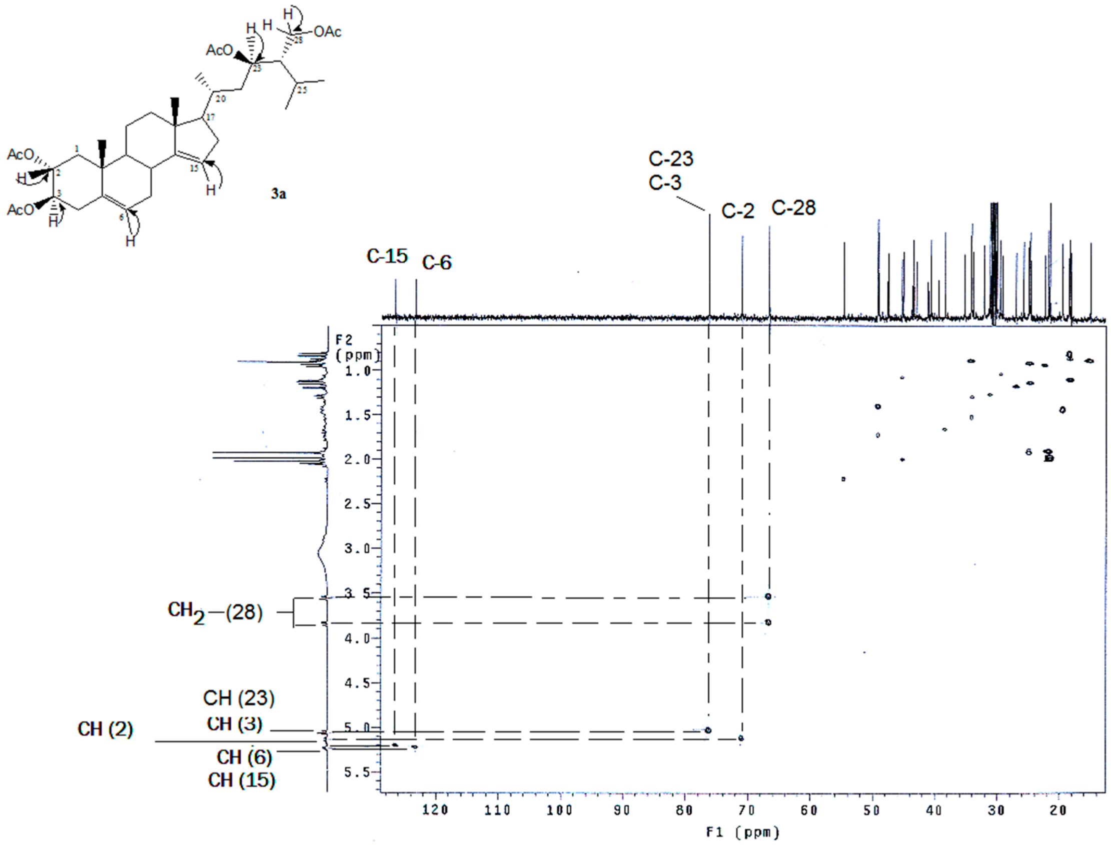


**Figure S4.** HSQC (400 MHz; acetone-$d_6$) of compound (23*R*)-2α,3β,23,28-tetraacetyl-14,15-dehydrocampesterol (**3a**).

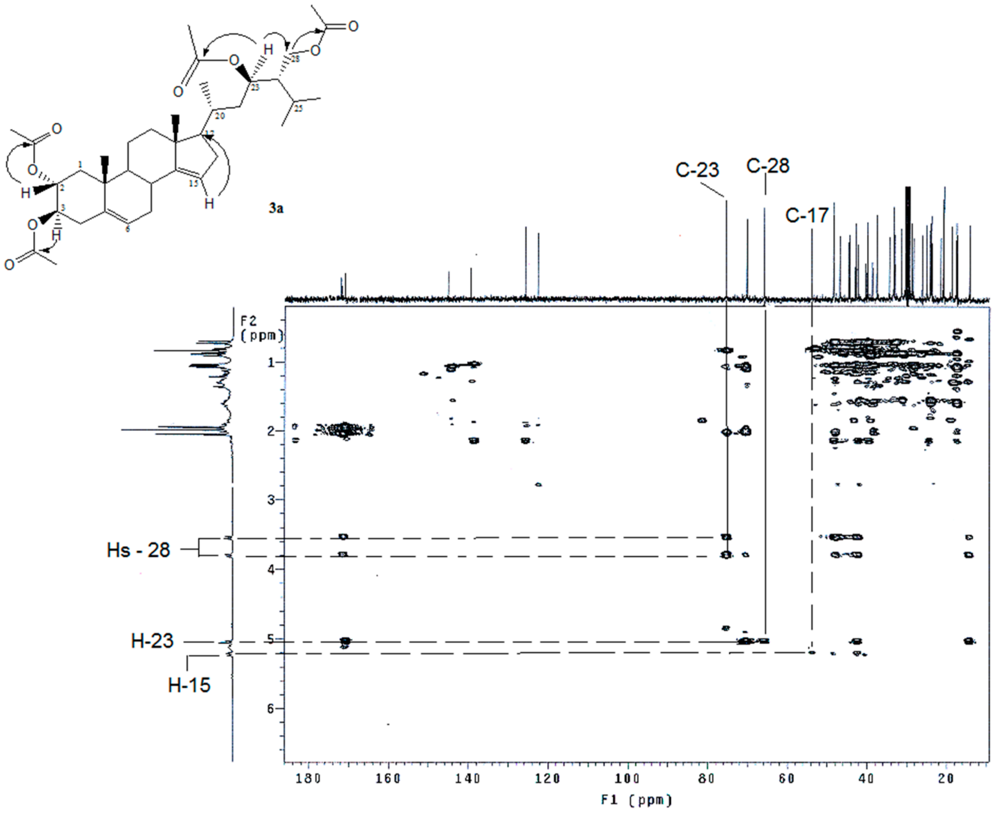


**Figure S5.** HMBC (400 MHz; acetone-$d_6$) of compound (23*R*)-2α,3β,23,28-tetraacetyl-14,15-dehydrocampesterol (**3a**).

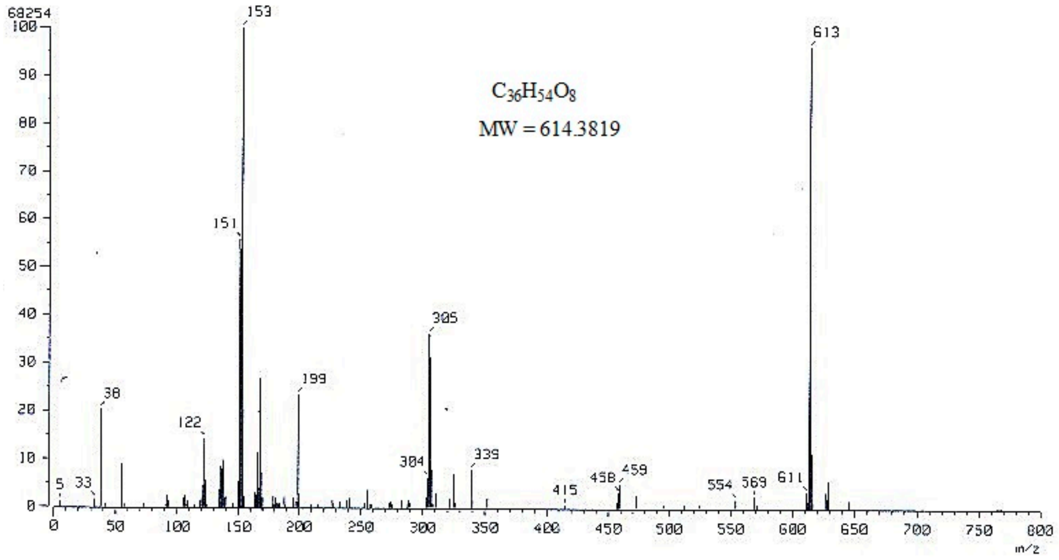


**Figure S6.** FABMS (negative).
